# Analysis in the Influencing Factors of Climate-Responsive Behaviors of Maize Growers: Evidence from China

**DOI:** 10.3390/ijerph19074274

**Published:** 2022-04-02

**Authors:** Hongpeng Guo, Yujie Xia, Chulin Pan, Qingyong Lei, Hong Pan

**Affiliations:** College of Biological and Agricultural Engineering, Jilin University, 5988 Renmin Street, Changchun 130022, China; ghp@jlu.edu.cn (H.G.); xiayj1997@163.com (Y.X.); pancl@jlu.edu.cn (C.P.); leiqingyong@sina.com (Q.L.)

**Keywords:** multi-variate Probit model, Poisson regression model, agricultural productive services

## Abstract

Due to the natural production properties, agriculture has been adversely affected by global warming. As an important link between individual household farmers and modern agriculture, it is crucial to study the influence of agricultural productive services on farmers’ climate-responsive behaviors to promote sustainable development and improve agricultural production. In this paper, a questionnaire survey has been conducted among 374 maize farmers by using the combination of typical sampling and random sampling in Jilin Province of China. Moreover, the Poisson regression and the multi-variate Probit model have been used to analyze the effects of agricultural productive services on the choices of climate-responsive behaviors as well as the intensity of the behaviors. The results have shown that the switch to suitable varieties according to the frost-free period have been mostly common among maize growers in Jilin province. Agricultural productive services have a significant effect on the adoption intensity of climate- responsive behaviors, at the 1% level. Based on this conclusion, this paper proposes policy recommendations for establishing a sound agricultural social service system and strengthening the support for agricultural productive services. It has certain reference significance for avoiding climate risk and reducing agricultural pollution in regions with similar production characteristics worldwide.

## 1. Introduction

Climate change has become a serious constraint on social and economic development. Many studies have zoomed into this issue in agriculture area, which is highly dependent on climate-sensitive resources [1,2]. Climate change will intensify the existing pressure on land and water resources. Due to the vulnerability of agriculture, climate change will affect poverty levels, especially those in Africa and Asia [1,3,4]. As a country with a population of 1.4 billion, food security is a crucial part of the national livelihoods of China. It is necessary to study the impacts of climate change on agricultural production and hence to come up with corresponding strategies to adapt to climate change to ensure food security [5,6]. The mode of agricultural production affects the severity of climate change in the future. Therefore, reducing greenhouse gas emissions in agriculture will mitigate the degree of climate change. The purpose of climate-smart agriculture is to solve the problem of poverty and food security under climate change. At the same time, it will mitigate the impacts of climate change on agricultural production [7,8]. The popularity of climate-smart agriculture has led to a growing number of the related literatures. Climate-smart agriculture has three major objectives: to increase agricultural yields, to enhance adaptation to climate change, and to mitigate greenhouse gas emissions [5,9,10,11]. Therefore, the analysis of the influencing factors of farmers’ low-carbon production adaptation practice plays an important role in achieving the goal of climate-smart agriculture.

In response to climate change, farmers will adopt some adjustments in agricultural production to avoid risks and to maintain the ecological environment, including low-carbon production practice and climate adaptation practice. Emission reduction refers to a series of measures to reduce the greenhouse gas emissions in agricultural production without affecting the economic efficiency of agricultural production. Through certain technologies, the material inputs and agricultural pollution have been reduced while ensuring agricultural production so as to achieve the joint development of economic, social and ecological benefits [12,13,14]. At present, scholars have conducted relevant studies and they have found that gender, age, and education level are governing factors affecting the low-carbon production practice [15,16]. Among household characteristics, family income, household size, and family business scale affect farmers’ low-carbon production practice greatly [16,17,18,19]. Moreover, policy and socio-cultural level also have certain impacts on farmers’ low-carbon production practice and fiscal incentives affect farmers’ emission reduction behaviors [20,21,22]. Social norms affect farmers’ soil conservation behaviors while the cost of low-carbon production practice affects farmers’ willingness for adaptation [16]. Farmers’ concerns for the environment and their technological awareness have certain impacts on farmers’ emission reduction behaviors. When considering promoting farmers’ emission reduction behaviors, farmers should be allowed to fully understand the costs and the risks of changing agricultural production methods [22,23,24,25,26]. Technical guidance and social service will reduce farmers’ resistance to low-carbon production practice, therefore promoting farmers’ low-carbon production behaviors [5,22,27,28]. The promotion of low-carbon agricultural production practice can effectively reduce greenhouse gas emissions, thus realizing the goal of climate-smart agriculture [14,22].

Adaptation is a deliberate change process of farmers themselves, which can cope with various pressures and changes that affecting people’s lives, to reduce the vulnerability of agriculture and improve its resilience [22,29]. Climate adaptation practice refers to the process of coping with the impacts of known or unknown climate change on agriculture by adjusting agricultural production mode [30]. Farmers are the microscopic main body of agricultural production and they are also the smallest decision-making unit for implementing climate adaptation practice. When farmers encounter or anticipate climate change during agricultural production, they will take corresponding measures to reduce agricultural production losses to maintain their normal yields. Past survey has shown that farmers will make corresponding changes in response to climate change [31]. Family characteristics also have significant impacts on the intensity of farmers’ climate adaptation practice and it is reflected in the form of human capital, social capital, economic capital and natural endowments. There’s a positive correlation shown between climate adaptation practice and family size, household income and relationship with neighbors [32]. There is also a positive correlation between climate adaptation practice and ecological environment [32]. The readiness of technology and resources and the availability of information affect farmers’ climate adaptation practice deeply [29,32]. Therefore, the level of technical service is a key factor affecting farmers’ climate adaptation practice [33]. The climate-adaptive behavior of farmers is also affected by pressure and institutions [29,34,35].

According to previous studies, climate-responsive behaviors can reduce agricultural production losses, maintain food security. Meanwhile, it can increase farmers’ income and promote stable global food production. It helps realize the goal of climate-smart agriculture. These behaviors are a form of “sustainable intensification”, which is an approach of agriculture that increases production without increasing adverse effects on the environment [36]. In this paper, climate response behaviors are divided into two categories: one is the low-carbon practice actively implemented by farmers, which is generally due to farmers’ awareness of the deterioration of the natural environment, and the purpose of which is to reduce agricultural pollution and agricultural emissions. The second is the climate adaptation practice that is passively implemented by farmers. These behaviors are the changes in production behaviors that farmers have to make due to climate change and climate disasters. Studying and evaluating the influencing factors of climate-responsive behaviors will help achieve the goals of climate-smart agriculture. Farmers are the main research object in this paper. Exploring the degrees of influence of individual characteristics, family characteristics, social characteristics, and access to productive agricultural services on farmers’ behaviors will help in the establishment of related policies around the world. They can take locally targeted measures to protect agro-ecosystems, mitigate climate change and increase yields. Moreover, it will drive the development of climate-smart agriculture, contributing to the realization of modern agricultural goals and agricultural sustainability [37].

### 1.1. Theoretical Analysis and Research Hypothesis

#### 1.1.1. Theoretical Analysis

The continuous global climate change will intensify the risks related to farmers’ agricultural production. As a rational economic entity, farmers will adopt various measures to ensure the yields and the product quality to maximize their benefits. At present, there are few studies on the impacts of agricultural productive services on farmers’ climate-responsive behaviors. However, there is a lack of systematic theoretical support for it. Therefore, we can treat the climate-responsive behaviors due to climate change as the farmers’ technology adoption behaviors. Hence the theory of farmers’ technology adoption behaviors can be used to analyze the adoption of farmers’ climate-responsive behaviors.

Drawing on the research ideas of A. Saha and other scholars, this paper constructs the decision model of farmers’ climate-responsive behaviors below [38].

(1) $$maxH=E_{i^{*}}\left[U(\tilde{W)}\right]=E_{i^{*}}\left\{U\left[p\left(f(m\right)+g\left(z\right)\tilde{e})-w\left(m+z\right)-rz\right]\right\}\;m+z=x,\tilde{Q}=f\left(m\right)+g\left(z\right)\tilde{e}$$

In this formula: H represents the net income of farmers, $E_{i^{*}}$ represents the income expectation of farmers when the amount of information is $i^{*}$, U represents the income function, $\tilde{W}$ represents a series of factors that affect the net income of farmers, $\tilde{Q}$ means that when there are m units of arable land area without climate-responsive behaviors and z units of arable land area adopting climate-responsive behaviors, where the total output of crops is represented by m+z=x. x is the total arable land area. Generally, the risks of farmers adopt climate-responsive behaviors is greater than the risks when they do not adopt climate-responsive behaviors. So, when climate-responsive behaviors are not adopted, the production function is non-random $f\left({}^{\circ}\right)$. When the climate-responsive behaviors are adopted, the production function is $g\left(z\right)\tilde{e}$ and $\tilde{e}$ is a random variable. w represents the costs incurred by farmers when climate-responsive behaviors are not adopted, r represents the additional costs incurred by farmers when adopting climate-responsive behaviors, and p represents the product price.

Since the main objective is to maximize benefits, whether to adopt climate-responsive behaviors depends on if there’s any changes to their expected benefits. With other conditions and factors remain unchanged, if the expected benefits of the farmers when adopting climate-responsive behaviors are greater than that of not adopting climate-responsive behaviors, they will make the adoption for their own good. The conditions for the farmers to adopt the climate-responsive behaviors are below:

(2) $$p_{1}g\left(m\right)\tilde{e}\left(Z\right)-\left(w+r\right)m\geq p_{0}f\left(m\right)-wm$$

$g\left({}^{\circ}\right)$ is the production function after the climate-responsive behaviors are adopted. $p_{1}$ represents the price of crops after the climate-responsive behaviors are adopted. $p_{0}$ represents the crop price when the climate-responsive behaviors are not adopted. *m* is the decision scale. $f\left({}^{\circ}\right)$ is the production function without the climate-responsive behaviors. $\tilde{e}\left(Z\right)$ represents the subjective risk function which is determined by Z. Z is a factor that affects decision-making and $\tilde{e}\left(Z\right)\in \left[0,1\right]$. The price of crops changes little before and after the farmers adopt the climate-responsive behaviors, so it can be assumed that $p_{0}=p_{1}$, then,

(3) $$\tilde{e}\left(Z\right)\geq \frac{p_{0}f\left(m\right)+rm}{p_{0}g\left(m\right)}$$

Price, production function and costs are relatively easy to determine in the above formula, while the subjective risk function $\tilde{e}\left(Z\right)$ is difficult to be determined. It depends on the adoption of agricultural productive services and the ambient environment in which they are located at. Hence, we can change the mathematical expression to:

(4) $$\tilde{e}\left(Z\right)=F\left\{G\left(I\right),H\left(O\right)\right\}$$

In (4), $G\left(I\right)$ indicates the resource endowment that affect farmers’ adoption of climate-responsive behaviors, including but not limited to personal characteristics, family characteristics, and social capital. $H\left(O\right)$ indicates other external factors that affect farmers’ adoption of climate-responsive behaviors, such as the adoption of agricultural productive services. The climate-responsive behaviors of farmers are affected by both.

#### 1.1.2. Research Hypothesis

##### Individual Characteristics of Farmers

Studies have shown significant differences between male and female farmers in terms of technology adoption in agricultural production [39]. At present, most agricultural production have been carried out by women. Compared with male farmers, females know better about how to take corresponding actions or measures to make adjustments for their agricultural production [33]. These measures help reduce the economic losses caused by climate change. However, other studies have also shown that, due to the influence of traditional concepts, women receive fewer resources compared with men, leading to the fact that women have been less empowered to make adjustment in their farming practice [15]. In Northeast China, men are still in the dominating position in their households. They make more decisions in practice, and they have more resource advantages. So, the male farmers in Northeast China tend to adopt climate-responsive behaviors in agricultural production. Some studies have shown that older people have more experience in terms of adjustments of agricultural production. As a result, these adjustment are more flexible, and their climate-responsive behaviors are more practical [33,40]. However, other studies have also shown that the age factor has a negative correlation with the adoption of new technology [40]. Education level can improve farmers’ ability to deal with climate change and adopt new technology used in future agricultural production [1,16,29].

**Hypothesis** **1.** *In comparison with females, male farmers are more likely to adopt climate-responsive behaviors. Age, experience, and education level have significant positive impacts on climate-responsive behaviors*.

##### Characteristics of Farmer Households’ Management

Farmer households with more family members can provide more labor resources for agricultural production. At the same time, their family members can do non-agricultural works, which will increase their overall family income and help relieve monetary pressure. This ensures better planning and conduction of agricultural production [17,38,41]. Moreover, households with more family members use more conservation tillage methods, and they tend to change more often on fertilizer usage [32,42]. Farmers with larger planting areas are generally more professional; hence they are more likely to adopt new technology [38]. Social capital can accelerate the delivery of information, which helps promote of agricultural technology adoption. In the meantime, it can also increase opportunities for collaboration and the successful application of loans. As a result, social capital can improve farmers’ awareness of climate, thereby affecting their climate-responsive behaviors [32].

**Hypothesis** **2.** *Household size, planting areas, and social capital have a significant positive impact on the adoption of climate-responsive behaviors*.

##### Government Subsidies and Loans

Different policy backgrounds affect farmers’ perception of climate change differently, and available subsidies from the government can reduce farmers’ monetary pressure, increase their production motivation and enhance farmers’ cooperation. This can help reduce the operating costs incurred, and farmers will be more likely to change their corresponding planting behaviors under climate change [20,22,23,25,35]. Loans can also reduce the monetary pressure farmers face in the agricultural production process, helping them to adopt new technology faster. Moreover, it encourages farmers to easily purchase fertilizers, pesticides, and new planting varieties. At the same time, it increases the possibility of improving the basic infrastructures of agricultural production. Generally, the availability of agricultural loans has a positive correlation with climate-responsive behaviors [32].

**Hypothesis** **3.** *Government subsidies and loans have a significant positive impact on the adoption of climate-responsive behaviors*.

##### Agricultural Productive Services

Agricultural productive services can assist agricultural production at all stages, from pre-production, production to post-production. It makes agricultural production activities more professional and well structured. At the same time, it also improves agricultural production efficiency greatly. Moreover, agricultural productive services improve labor-shortage problems, introduce advanced agricultural production technology to farmers [43,44]. It can improve farmers’ understanding of climate change and increase the intensity of farmers’ climate-responsive behaviors [32]. Furthermore, agricultural productive services can increase farmers’ awareness of advanced technology, helping them adopt low-carbon production practices easily. As a result, the promotion of related production behaviors can be improved [5,15,22,26,27,28].

**Hypothesis** **4.** *Agricultural productive services have a positive impact on the adoption of climate-responsive behaviors*.

## 2. Materials and Methods

### 2.1. Model Setting

#### 2.1.1. Poisson Regression

When farmers perform climate-responsive behaviors, there will be differences in their behaviors’ intensity. The number of behaviors implemented is not a binary variable, whereas it is a limited dependent variable that takes only positive integer values. Moreover, the dependent variable is the sum of the types of farmers’ climate-responsive behaviors, which is a counting variable. Linear models are not suitable. For such dependent variables, the Poisson distribution has been chosen [45,46].

$$P\left(Y_{i}=y_{i}|x_{i}\right)=\frac{e^{-\lambda_{i}}\lambda_{i}^{y_{i}}}{y_{i}!}\left(y_{i}=0,1,2,\cdots \right)$$

Since the number of behaviors is a counting variable, such discrete variables obey the Poisson distribution. The model can be set as below:

For individual i, this article assumes that the explained variable is $Y_{i}$. $\lambda_{i}>0$, which is the “Poisson arrival rate”, representing the average number of events. It is determined by $x_{i}$. The expectation and variance of the variable distributions are the same in the Possion regression and they are equal to the Poisson arrival rate. In order to ensure that the Poisson arrival rate is negative, this paper makes the assumptions:

$$E\left(Y_{i}|x_{i}\right)=\lambda_{i}=\exp\left(x_{i}^{\prime }\beta \right)$$

In this way, the intensity of the farmers’ climate-responsive behaviors can be linked to the explanatory variables. In this paper, the Poisson regression has been used to calculate the impacts of farmers’ use of productive services on the intensity of the implementation of climate-responsive behaviors.

#### 2.1.2. Multi-Variate Probit Model

The Probit model is a discrete model and generally it’s used to fit a 0−1 dependent variable regression. ε is an error term that obeys the standard normal distribution [47].

Ordinary multiple linear regression equation:

$$Y=\beta_{0}+\overset{n}{\underset{i=1}{\sum }}\beta_{i}x_{i}+\varepsilon \;E\left(y|x\right)=1\cdot P\left(y=1|x\right)+0\cdot P\left(y=0|x\right)=P(y=1|x)$$

If $F\left(x,\beta \right)$ is the standard normal cumulative distribution function:

$$P\left(y=1|x\right)=F\left(x,\beta \right)=\Phi \left(x^{\prime },\beta \right)=\int_{-\infty }^{x\prime \beta }\varphi \left(t\right)dt$$

When studying the choice of n types of production behaviors of farmers, it is necessary to estimate the n Probit models. The implicit assumption is that the error term ε between the n Probit models are not related to each other. However, in actual agricultural production, farmers may choose a variety of climate-responsive behaviors and these behaviors are not mutually exclusive [48]. Therefore, some variables that cannot be observed may affect farmers to adopt different climate-responsive behaviors at the same time. The above-mentioned error term may be correlated. Therefore, this article uses a multi-variate Probit model for the estimation. It contains multiple binary dependent variables:

$$y_{im}^{*}=\alpha_{i}S+\beta_{i}Control+\varepsilon_{im}\;y_{im}=\left\{\begin{array}{l} 1,\;if\;y_{im}^{*}>0\\ 0,\;x \end{array}\right.$$

Among them, $y_{im}^{*}$ is a potential variable for the individual i to implement m kinds of climate-responsive behaviors. m=1,2,3,4,5,6,7, indicate the adoption of planting-breeding combination methods, the conservation tillage methods, the switch-over to organic fertilizers, the change of suitable varieties according to the frost-free periods, the adjustment of pesticides and fertilizer usage, the adjustment of the time of sowing and harvesting, and the replenishing seedlings, respectively. $y_{im}$ is the final result variable and $\varepsilon_{im}$ is a random disturbance term, which obeys a multi-variate normal distribution with a mean value of 0 and a covariance of K, $\varepsilon_{im}\sim \left(0,k\right)$.

### 2.2. Data Sources

Data used in this article comes from questionnaire surveys conducted on maize farmers by the research team from June to September in 2021 in Jilin province in Northeast China. Based on existing documents and interviews with farmers, combined with the characteristics of agricultural production in Jilin province. The area has an average annual rainfall of 400–800 mm, 3000 h of sunshine and high-quality soil. The farmers come from Changchun, Songyuan, Jilin, Siping and Liaoyuan cities in Jilin Province. This area is characterized by rain hot during the same period. It is flat, fertile and has four distinct seasons, making it a good place to grow corn in China. Questionnaires on agricultural productive services and climate-responsive behaviors have been made. It includes the basic information of farmer households, the adoption of agricultural productive services, and their awareness of climate-responsive behaviors. A combination of typical sampling and random sampling has been used for sample selection. A total of 389 questionnaires have been conducted with, 374 of them were being valid. The overall effective rate was 96%.

### 2.3. Variable Setting and Descriptive Statistics

To determine the impacts of agricultural productive services on climate-responsive behaviors, it is necessary to determine what climate-responsive behaviors are. There are various types of low-carbon practice and climate adaptation practice. However, not all of these methods are suitable for the farmers in Jilin province of China. After literature review and in-person interviews, related information has been obtained. At present, the main low-carbon production practice aiming to reduce agricultural pollution used by farmers in Jilin province includes planting-breeding combination methods, conservation tillage methods, and organic fertilizers. Climate adaptation practice aiming at reducing yield losses and increasing profits includes the change of suitable varieties according to the frost-free periods, the adjustment of pesticides and fertilizer usage, the adjustment of the time of sowing and harvesting, and the filling of gaps with seedlings.

**Figure 1:**
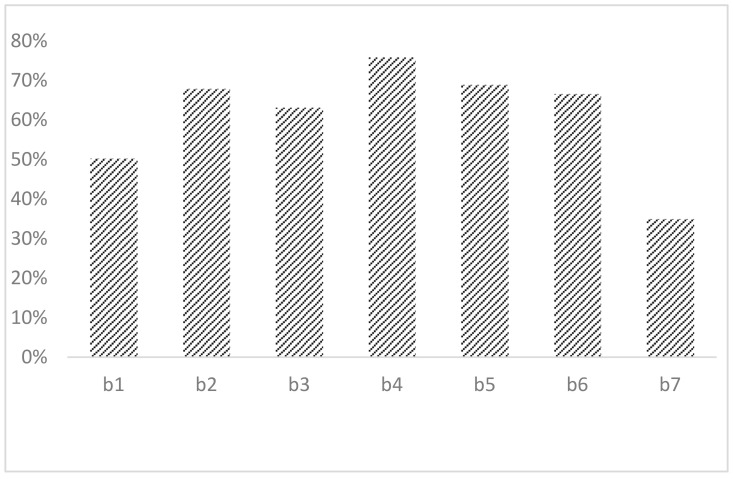
Farmers’ behaviors.

Figure 1 shows the adoption of farmers’ climate-responsive behaviors. From the left to the right: planting-breeding combination methods (b1), conservation tillage methods (b2), use of organic fertilizers (b3), the change of suitable varieties according to the frost-free periods (b4), adjustment of pesticides and fertilizer usage (b5), adjustment of the time of sowing and harvesting (b6), filling the gaps with seedlings (b7), respectively.behaviors. Among the farmers being surveyed, changing suitable planting varieties according to the frost-free periods is the most used method, followed by adjusting pesticides and fertilizers usage. Conservation tillage is commonly used in low-carbon production practice, which also shows that the promotion of conservation tillage is effective.

The climate-responsive methods adopted by the farmers will be assigned with a value of 1, otherwise it is set to be 0. When performing multi-variate Probit test, regression is performed on each behavior separately. When performing the Poisson regression, the dummy variables of corresponding variables are summed. The more types of method being used, the higher the intensity of climate-adaptive behaviors would be.

Based on the hypothesizes made in Section 2.2, a total of 9 variables in 4 aspects namely farmers’ individual characteristics, farmer households’ characteristics, government subsidies and loans, and agricultural productive services are selected for analysis. Detailed definitions and explanations of the variables can be seen in Table 1.

## 3. Results and Analysis

With the survey data obtained, the nine independent variables are tested for multicollinearity before regression. It is showed in Table 2 that the variance inflation factor (VIF) value of all variables is less than 10 and the average value is only 1.13. It means that the selected explanatory variables all meet the principle of independence, so there is no multicollinearity problem anticipated and regression analysis can be performed.

### 3.1. Analysis of the Factors Affecting the Intensity of Low-Carbon Production Practice and Climate Adaptation Practice

Climate response behavior is divided into low-carbon production practice aiming at protecting the environment and mitigating climate change and climate adaptation practice aiming at reducing climate-induced agricultural production loss. The influencing factors of low-carbon production behavior intensity and climate adaptation behavior intensity were analyzed, respectively. Stata15.1 software (Statacorp LLC, College Station, TX, USA) has been used to perform the Poisson regression on the survey data collected, and Table 3 shows that the overall fitting effect is good. Table 3 also shows the estimation results of the general least squares (OLS). No matter which model is used, the regression is significant overall, and the significance of the estimated coefficients is the same.

Gender is significant at the 1% level in the adoption of low-carbon production practice and climate-responsive practice, and it has a remarkable negative impact on them. The probability of female farmers adopting low-carbon production behaviors is 25.4% lower than that of male farmers. The probability of female farmers adopting climate adaptation practice is 16.0% lower than that of men. With other conditions unchanged, men tend to adopt climate-responsive behaviors compared with women farmers. This shows that women are generally not willing to change their agricultural production behaviors, hence changes in production behaviors caused by climate are not as good as men. Influenced by traditional concepts, men, as the dominant force in family production, receive better resources than women and men are more inclined to switch to new production behaviors. In this part, hypothesis 1 was verified. Age has an insignificant influence on the intensity of climate-responsive behaviors, so the hypothesis is not valid. Education level has a significant positive impact on farmers’ low-carbon production practice. With each education level up, the possibility of adopting low-carbon production behaviors increases by 12.2%. This shows that those who have higher education level has a stronger awareness of global warming. At the same time, they are more inclined to emission reduction and they tend to adopt low-carbon production behaviors more compared with the rest. It’s also worth to mention that education level has an impact on climate adaptation practice although it’s not significant. With other conditions remain unchanged, farmers with higher education levels are more cognizant and they are more inclined to adopt climate-responsive behaviors. People with higher education levels are more likely to learn new production techniques and they often understand the climate better.

Household size and planting area do not significantly affect the intensity of climate-responsive behaviors. In Northeast China, families with more family members are generally old-fashioned with average or below-average household income. Hence, these farmers are not sensitive to climate-responsive agricultural production behaviors.

The impact of planting area on climate-responsive behaviors is not significant, and the hypothesis cannot be verified.

Government subsidies have positive impacts on climate adaptation practice and it is significant at the 5% level. At the same time, the impact of government subsidies and loans are not significant on low-carbon production practice. Government subsidies can reduce catastrophic losses caused by climate change and reduce the costs incurred during agricultural production. So, farmers are more inclined to carry out technological transformation. Loans have an insignificant negative correlation with the adoption of climate-responsive behaviors.

Agricultural productive services have a clear positive impact on climate-responsive behaviors. The impact of agricultural productive services on the intensity of climate adaptation practice and climate adaptation practice are both significant at the 1% level in both OLS and Poisson. The possibility of adopting low-carbon production practice increases by 7.8%, and that of adopting climate-adaptive services increases by 9.4% for each additional productive service used by farmers. The adoption of agricultural productive services helps to achieve the emission reduction goal advocated by climate-smart agriculture. It improves the level of farmers’ adaptive behaviors at the same time. Agricultural productive services enable farmers to obtain climate change information promptly. The agricultural production technology level of farmers continues to improve, and the relevant agricultural materials can be obtained by farmer households faster. The entire agricultural activities are more specialized and properly divided, and the efficiency of agricultural production is improved. Farmers tend to adopt flexible production methods to promote the occurrence of climate response behaviors.

### 3.2. Heterogeneity Analysis

The result in Table 3 describes how various variables influence the climate-responsive behaviors. Farmers’ adoption of climate-responsive behaviors is significantly affected by productive services. To exploit the difference between the influence brought by productive services, this part does analysis from three aspects: age, gender, and planting scale.

#### 3.2.1. Age

With the continuously growing social development in recent years, generation gap has become a more critical influencing factor in the ways of thinking of individuals. Similarly, this effect can be observed when people accept fresh viewpoints, adopt agricultural productive services and put theoretical knowledge into practices. After conducting research in Chinese rural regions, the farmer in this paper can be categorized into three types: the young generation (born after 1980), the middle-age generation (born between 1965 and 1979), and the old generation (born before 1965) [49].

According to Table 4, after controlling the variable, the young and the middle-age are more tendentious to adopt low-carbon production practice and climate adaptation practice under the influence of agricultural productive services. Oppositely, the minor response in the old indicates that the willingness of elder farmer on learning and adopting advanced technologies, and they have less tendency on changing the conventional methods they used.

#### 3.2.2. Gender

The analysis conducted in Section 2.2 is based on two groups, female and male. According to Table 5, the level of female farmers who adopt climate-responsive behaviors is not remarkably influenced by the extent of they accept productive services. However, a significant feedback can be found in the analysis of another group. Therefore, it seems that agricultural productive services have brought great impact to male farmers’ adoption of climate-responsive behaviors. At the same time, the analysis tells that the attitude of the females are more conventional when embracing advanced technologies. The males, on the contrary, are more likely to improve the production efficiency by changing technical practices.

#### 3.2.3. Planting Scale

In the third national agricultural census, the planting area in the region where is one crop per annum is categorized into two types: small scale (6.7 hectares) and large scale (greater than 6.7 hectares) [50]. The data, according to this regulation, is separated into the small-scale farmer and the large-scale farmer group. Analyses are made independently in two groups to see the impact of agricultural productive services on farmers’ adoption of climate-responsive behaviors.

The result in Table 6 describes that the productive services increase the acceptance of climate-responsive behaviors in both groups. However, the likelihood of those farmers who have a smaller scale planting area is relatively low. Individuals in the large-scale farmer group trust more in local technicians and agricultural extension personnel. At the same time, they earn more attention from the local government. Hence, they have more opportunities to attend training activities in agricultural technologies, and they are more likely to accept advanced knowledge, which develops the level of climate-responsive behaviors.

### 3.3. Analysis of Factors Affecting Farmers’ Choices for Climate-Responsive Behaviors

Reference to Stata 15.1, a multi-variate Probit model has been used to estimate the choices of climate adaptation practice of the maize farmers in Jilin province. In the correlation matrix, 19 correlation coefficients ρ are significant at the 1% level and all correlation coefficients are significant at the 5% level. This result shows that, farmers’ choices of climate-responsive behaviors are influenced by other climate-responsive behaviors. The planting-breeding combination methods, the conservation tillage methods, the use of organic fertilizers, the changes of planting varieties according to the frost-free periods, the adjustment of using pesticides and fertilizers, the adjustments of sowing and harvesting time and the filling of the gaps with seedlings are complementary to one another. The probability of using other methods will grammatically increase after adopting a single climate-responsive method. Table 7 shows the result of parametric regression and the model is significant at all levels. Detailed explanations of the result according to different variables are discussed as follows:

Gender is significant at the 1% level in the adoption of the planting-breeding combination methods, the conservation tillage methods and the changing of planting varieties according to the frost-free periods’ methods. It is significant at the 5% level in the adoption of using organic fertilizers and the adjustment of fertilizers and pesticides usage. It is significant at the 10% level in the adoption of filling the gaps with seedlings.

Age has a remarkable positive impact on the changing of planting varieties according to the frost-free periods’ methods, the adjustment of pesticides and fertilizers usage and the adjustment of sowing and breeding time. These three climate-responsive behaviors are the most empirical ones among the seven behaviors discussed in this article. Farmers who have been working in the agricultural production industry for a long time can adjust on the basis of their previous experiences. Education level is significant at the 1%, 5%, 10% level in the adoption of the planting-breeding combination methods, the usage of organic fertilizers and the adjustment of the time of sowing and breeding, respectively. At the same time, education level has a significant positive impact on these three climate-responsive behaviors.

Social capital has a 10% significance in the method of switching over to organic fertilizers and the adjustment of sowing and harvesting time.

Government subsidies are significant at the 5% and 10% level in the adjustment of the pesticides and fertilizers usage and the filling of the gaps with seedlings, respectively. This shows that, government subsidies help reduce farmers’ monetary pressure and the related risky behaviors in great deal. This is especially obvious in the filling of the gaps with seedlings behaviors.

Agricultural productive services have positive impacts on climate-responsive behaviors. It is significant at the 1% level in the adoption of conservation tillage methods, the adjustment of sowing and harvesting time and replanting methods. Agricultural productive services also have positive impacts on the planting-breeding combination methods and the usage of organic fertilizers although it’s not significantly.

Agricultural productive services have positive impacts on climate response behaviors. Among them, the conservation tillage methods, the adjustment of pesticides and fertilizers usage, the adjustment of sowing and harvesting time and the behaviors of replenishing seedlings have significance at 1%.

## 4. Discussion

Based on the survey conducted on 374 maize farmers in Jilin province of China, an empirical analysis of the low-carbon production practice and climate adaptation practice due to climate change have been carried out, with findings.

Firstly, most maize farmers adjust their production methods promptly according to the changes of climate. Conservation tillage methods have been used the most by farmers among all the low-carbon production practice. Among all of the adaptation methods practiced, 75.9% of the farmers will choose suitable varieties according to the frost-free periods and 69.0% of the farmers will adjust their usage of pesticides and fertilizers according to different climate conditions. Secondly, gender and education level have a remarkable impact on the intensity of farmers’ climate-responsive behaviors. Thirdly, agricultural productive services have crucial impacts on farmers’ climate-responsive behaviors. This part validates the hypothesis. Lastly, in actual operation, farmers have used methods according to local needs.

## 5. Suggestion

Based on the data and the analysis conducted, policy recommendations related to the existing problems are proposed as below.

Firstly, agricultural socialization service system needs to be improved, different service suppliers need to be developed, agricultural market needs to be standardized, and the construction of market needs to be strengthened.

Secondly, the government should continue to support on the provision of information and technology related to agricultural production. The government can set up propaganda boards in the village, distribute leaflets, and provide guidance for technical personnel to enter the households, so that the villagers know its benefits and effects and increase the acceptance of agricultural productive services. Agricultural productive services need to be diversified. It will not only help with the realization of climate-smart agriculture and the adaptation of farmers to climate change, but will also help to reduce external risks during production processes.

Thirdly, as an essential and highly specialized portion, emission reductions can be outsourced to external business entities. It will also help to realize the goal of low-carbon production as those related technology does not need to be promoted to farmers.

On the fourth, farmers’ education level needs to be improved and the financial supports on farmers’ education need to be strengthened. These measures will help to improve the farmers’ understanding of climate change and their adaptive methods.

On the fifth, the availability and readiness of loans and subsidies for farmers should be improved. Risks coming from climate change can be avoided with the guidance and control of agricultural subsidies.

In general, agricultural productive services have a great impetus to farmers’ climate-responsive behaviors. Globally, agricultural production characteristics with similar climatic characteristics may have similar effects.

## 6. Conclusions

In contrast, the males are willing to try and use new ideas and technologies, while females tend to be conservative. This part validates the hypothesis. In addition, farmers with higher education level have stronger understanding and ability to accept new things. They have more positive attitudes towards environmental protection, and they are more likely to adopt climate-responsive behaviors. In previous literatures, some scholars have also verified the influencing factors of these factors. Their proof is similar to the conclusions of this study [15,16,48]. Agricultural productive services help households to participate in the division and proper allocation of labor. It helps farmers to obtain information related to climate change, government support on technology and resources promptly. Therefore, agricultural productive services have positive impacts on climate-responsive behaviors. This conclusion has also been confirmed by scholars in previous studies. They believe that training services and information disclosure can help rainfed farmers develop reasonable adaptation measures to reduce climate risks to agriculture [48,51]. Different farmer groups have different performances in agricultural productive services promoting climate-responsive behaviors. It has a more significant effect on farmers with male, large-scale planting and younger characteristics. Males are more receptive to new technologies than women. Younger farmers have stronger learning ability and can translate new ideas into practice well. Large-scale planting farmers are valued by local agricultural associations. They received more information and technical training, resulting in higher adoption of climate-responsive behaviors by farmers.

The method used in this paper fails to study the internal mechanism of agricultural productive services on farmers’ behaviors. In addition, the consistency of farmers’ willingness and behavior needs to be further explored. According to the effects of different types of agricultural productive services, a comparative analysis can be carried out to find the most effective method in the region. In the future, we can also discuss the specific relationship between gender and farmers’ behavior so as to contribute to promoting equality between males and females and solving social conflicts.

## Figures and Tables

**Table 1:** Definition and Descriptive Statistics of Variables.

Variables	Value	Mean Value	Standard Deviation	Min	Max
Gender	1 = Male, 2 = Female	1.353	0.479	1	2
Age	Age of farmers	46.385	7.5	28	68
Education level	1 = Primary school education and below, 2 = Junior high school education, 3 = High school education, 4 = University degree	2.414	0.766	1	4
Household size	Population of peasant households (people)	3.882	1.230	1	10
Planting area	Operating land area of peasant household (ha)	16.624	35.398	0.053	500
Social capital	Neighbors help each other during the busy period, 1 = Yes, 0 = No	0.882	0.323	0	1
Government subsidies	Receive agricultural subsidies, 1 = Yes, 0 = No	0.802	0.399	0	1
Loans	Take loans, 1 = Yes, 0 = No	0.537	0.499	0	1
Agricultural productive services	Number of agricultural productive services received, 1–5 (Agricultural resources service, 1 = Yes, 0 = No. Rural insurance service, 1 = Yes, 0 = No. Service of agricultural machinery, 1 = Yes, 0 = No. Hires labor, 1 = Yes, 0 = No. Technical training, 1 = Yes, 0 = No)	3.856	1.149	0	5

**Table 2:** Multicollinearity.

Variables	VIF
Gender	1.04
Age	1.18
Education level	1.21
Household size	1.08
Planting area	1.17
Social capital	1.07
Government subsidies	1.08
Loans	1.14
Agricultural productive services	1.19
Mean VIF	1.13

**Table 3:** Estimation of the intensity of low-carbon production practice and climate adaptation practice of farmers.

	Low-Carbon Production Practice			Climate Adaptation Practice		
	Poisson Regression	OLS	Incidence-Rate Ratios	Poisson Regression	OLS	Incidence-Rate Ratios
Gender	−0.293 ***	−0.499 ***	0.746	−0.175 ***	−0.417 ***	0.840
	(−4.51)	(−4.75)		(−3.21)	(−3.18)	
Age	0.046	0.093	1.047	0.183	0.483	1.201
	(−0.29)	(−0.33)		(−1.13)	(−1.36)	
Education level	0.115 ***	0.216 ***	1.122	0.043	0.108 *	1.044
	(−3.19)	(−3.05)		(−1.29)	(−1.23)	
Household size	0.003	0.008	1.003	−0.023	−0.046	0.977
	(−0.54)	(−0.39)		(−1.3)	(−1.70)	
Planting area	0.079	0.123	1.082	0.143	0.309	1.154
	(−0.90)	(−0.78)		(−1.46)	(−1.57)	
Social capital	0.000	0.000	1.000	−0.001	−0.002	0.999
	(−0.24)	(−0.15)		(−0.92)	(−0.87)	
Government subsidies	0.061	0.116	1.063	0.174 **	0.403 **	1.190
	(−0.80)	(−0.90)		(−2.43)	(−2.52)	
Loans	−0.049	−0.096	0.953	−0.022	−0.061	0.978
	(−0.84)	(−0.91)		(−0.42)	(−0.46)	
Agricultural productive services	0.0748 ***	0.129 ***	1.078	0.0896 ***	0.210 ***	1.094
	(−2.66)	(−2.75)		(−3.32)	(−3.60)	
_cons	0.280	1.247 ***	1.323	0.440 **	1.382 ***	1.552
	(−1.38)	(−3.27)		(−2.07)	(−2.91)	
N	374	374		374	374	
P	0.0000	0.0000		0.0000	0.0000	

OLS: Ordinary Least Square; Cons: constant terms; z statistics in parentheses; N sample size; * *p* < 0.1, ** *p* < 0.05, *** *p* < 0.01; Indicate that the coefficients of the explanatory variables are significant at the 10%, 5%, and 1% levels, respectively.

**Table 4:** Regression results of different generations of farmers.

**Low-Carbon Production Practice**	**The Young Generation**		**The Middle-Age Generation**		**The Old Generation**	
Agricultural productive services	1.1258 *** (2.04)		1.0713 *** (2.09)		0.9027 (−0.76)	
Control variable	Controlled		Controlled		Controlled	
Log likelihood	−128.90164		−371.99202		−41.994357	
N	93		251		30	
P	0.0000		0.0000		0.0230	
Pseudo R^2^	0.0645		0.0223		0.0245	
**Climate Adaptation Practice**	**The Young Generation**		**The Middle-Age Generation**		**The Old Generation**	
Agricultural productive services	1.1060 ***	2.93	1.0933 ***	2.81	0.0605	0.1370
Control variable	Controlled		Controlled		Controlled	
Log likelihood	−156.0848		−410.3360		−48.7649	
N	93		251		30	
P	0.0000		0.0032		0.0607	
Pseudo R^2^	0.0545		0.0223		0.0729	

z statistics in parentheses. N sample size. *** *p* < 0.01. Indicate that the coefficients of the explanatory variables are significant at the 10%, 5%, and 1% levels, respectively.

**Table 5:** Regression results of different gender.

**Low-Carbon Production Practice**	**Female**	**Male**
Agricultural productive services	1.0570 (0.97)	1.0828 *** (2.64)
Control variable	Controlled	Controlled
Log likelihood	−181.3164	−362.0998
N	132	242
P	0.0053	0.0095
Pseudo R^2^	0.0341	0.0129
**Climate Adaptation Practice**	**Female**	**Male**
Agricultural productive services	1.0829 (1.55)	1.0826 *** (2.62)
Control variable	Controlled	Controlled
Log likelihood	−208.8982	−409.3071
N	132	242
P	0.0000	0.0000
Pseudo R^2^	0.0473	0.0223

z statistics in parentheses. N sample size. *** *p* < 0.01. Indicate that the coefficients of the explanatory variables are significant at the 10%, 5%, and 1% levels, respectively.

**Table 6:** Regression results of different scale.

**Low-Carbon Production Practice**	**Small Scale**		**Large Scale**	
Agricultural productive services	1.0368	0.95	1.1019 **	2.41
Control variable	Controlled		Controlled	
Log likelihood	−293.7519		−252.1136	
N	199		242	
P	0.0002		0.0004	
Pseudo R^2^	0.0226		0.0314	
**Climate Adaptation Practice**	**Small Scale**		**Large Scale**	
Agricultural productive services	1.0658 * (1.70)		1.0861 ** (2.17)	
Control variable	Controlled		Controlled	
Log likelihood	−326.3521		−291.1609	
N	132		242	
P	0.0066		0.0003	
Pseudo R^2^	0.0271		0.0403	

z statistics in parentheses. N sample size. * *p* < 0.1, ** *p* < 0.05. Indicate that the coefficients of the explanatory variables are significant at the 10%, 5%, and 1% levels, respectively.

**Table 7:** Estimation of farmers’ choices of climate-responsive behaviors.

	Planting-Breeding Combination Methods	Conservation Tillage Methods	Use of Organic Fertilizers	The Change of Suitable Varieties according to the Frost-Free Periods	Adjustment of Pesticides and Fertilizers Usage	Adjustment of the Time of Sowing and Harvesting	Filling the Gaps with Seedlings
Gender	−0.540 ***	−0.560 ***	−0.294 **	−0.480 ***	−0.360 **	−0.204	−0.257 *
	(−3.81)	(−3.87)	(−2.08)	(−3.24)	(−2.5)	(−1.44)	(−1.8)
Age	0.000	−0.003	0.517	0.884 **	0.737 *	0.737 *	−0.233
	(0.00)	(−0.01)	(1.29)	(2.08)	(1.83)	(1.86)	(−0.58)
Education level	0.178 *	0.159	0.312 ***	0.048	0.095	0.412 ***	−0.099
	(1.81)	(1.52)	(3.05)	(0.45)	(0.92)	(3.84)	(−1.02)
Household size	0.063	−0.021	0.086	0.079	0.016	−0.018	−0.011
	(1.07)	(−0.35)	(1.44)	(1.21)	(0.27)	(−0.3)	(−0.19)
Planting area	−0.165	0.172	0.345 *	0.240	0.311	0.346 *	−0.024
	(−0.77)	(0.81)	(1.68)	(1.07)	(1.48)	(1.65)	(−0.11)
Social capital	0.003	−0.001	−0.002	−0.001	−0.001	0.001	−0.004
	(1.06)	(−0.32)	(−0.78)	(−0.39)	(−0.67	(0.37	(−1.57
Government subsidies	0.275	0.003	0.022	0.228	0.332*	0.162	0.406 **
	(1.56)	(0.02)	(0.13)	(1.26)	(1.9)	(0.92)	(2.23)
Loans	−0.046	−0.279 *	0.008	0.037	−0.192	−0.062	−0.024
	(−0.32)	(−1.85)	(0.06)	(0.24)	(−1.31)	(−0.43)	(−0.17)
Agricultural productive services	0.090	0.210 ***	0.082	−0.027	0.237 ***	0.207 ***	0.186 ***
	(1.42)	(3.21)	(1.3)	(−0.38)	(3.65)	(3.23)	(2.75)
_cons	−0.362	0.162	−1.184 **	0.257	−0.924	−1.702 ***	−0.605
	(−0.66)	(0.28)	(−2.08)	(0.43)	(−1.61)	(−2.97)	(−1.05)
N		374					
P		0.0000					
ρCorrelation coefficient matrix: ρ21 = 0.2786 *** ρ31 = 0.2479 *** ρ41 = 0.2907 *** ρ51 = 0.2926 *** ρ61 = 0.3155 *** ρ71 = 0.3043 *** ρ32 = 0.1273 ** ρ42 = 0.1758 *** ρ52 = 0.3688 *** ρ62 = 0.3387 *** ρ72 = 0.1325 ** ρ43 = 0.1788 *** ρ53 = 0.2539 *** ρ63 = 0.2100 *** ρ73 = 0.3059 *** ρ54 = 0.2581 *** ρ64 = 0.2774 *** ρ74 = 0.2035 *** ρ65 = 0.4562 *** ρ75 = 0.1773 *** ρ76 = 0.2826 ***							
Wald χ^2^		140.13					
Log likelihood		−1411.93					

OLS: Ordinary Least Square. Cons: constant terms. z statistics in parentheses. N sample size. * *p* < 0.1, ** *p* < 0.05, *** *p* < 0.01 indicate that the coefficients of the explanatory variables are significant at the 10%, 5%, and 1% levels, respectively.

## Data Availability

Not applicable.

## References

[1] Le Dang H., Li E., Nuberg I., Bruwer J. (2019). Factors influencing the adaptation of farmers in response to climate change: A review. Clim. Dev..

[2] Karki S., Burton P., Mackey B. (2019). The experiences and perceptions of farmers about the impacts of climate change and variability on crop production: A review. Clim. Dev..

[3] Shirsath P.B., Aggarwal P., Thornton P., Dunnett A. (2017). Prioritizing climate-smart agricultural land use options at a regional scale. Agric. Syst..

[4] Mahmood N., Arshad M., Kächele H., Ma H., Ullah A., Müller K. (2019). Wheat yield response to input and socioeconomic factors under changing climate: Evidence from rainfed environments of Pakistan. Sci. Total Environ..

[5] Long T.B., Blok V., Coninx I. (2016). Barriers to the adoption and diffusion of technological innovations for climate-smart agriculture in Europe: Evidence from the Netherlands, France, Switzerland and Italy. J. Clean. Prod..

[6] Regan P.M., Kim H., Maiden E. (2019). Climate change, adaptation, and agricultural output. Reg. Environ. Chang..

[7] Kpadonou R.A.B., Owiyo T., Barbier B., Denton F., Rutabingwa F., Kiema A. (2017). Advancing climate-smart-agriculture in developing drylands: Joint analysis of the adoption of multiple on-farm soil and water conservation technologies in West African Sahel. Land Use Policy.

[8] Ngoma H., Lupiya P., Kabisa M., Hartley F. (2021). Impacts of climate change on agriculture and household welfare in Zambia: An economy-wide analysis. Clim. Chang..

[9] Andrieu N., Sogoba B., Zougmore R., Howland F., Samake O., Bonilla-Findji O., Lizarazo M., Nowak A., Dembele C., Corner-Dolloff C. (2017). Prioritizing investments for climate-smart agriculture: Lessons learned from Mali. Agric. Syst..

[10] Arslan A., McCarthy N., Lipper L., Asfaw S., Cattaneo A., Kokwe M. (2015). Climate Smart Agriculture? Assessing the Adaptation Implications in Zambia. J. Agric. Econ..

[11] Tong Q., Swallow B., Zhang L., Zhang J. (2019). The roles of risk aversion and climate-smart agriculture in climate risk management: Evidence from rice production in the Jianghan Plain, China. Clim. Risk Manag..

[12] Grottera C., La Rovere E.L., Wills W., Pereira A.O. (2020). The role of lifestyle changes in low-emissions development strategies: An economy-wide assessment for Brazil. Clim. Policy.

[13] Hamdi-Cherif M., Li J., Broin E. (2021). Leveraging the transport sector to mitigate long-term climate policy costs in China: A behavioural perspective. Clim. Policy.

[14] Harada H., Kobayashi H., Shindo H. (2007). Reduction in greenhouse gas emissions by no-tilling rice cultivation in Hachirogata polder, northern Japan: Life-cycle inventory analysis. Soil Sci. Plant Nutr..

[15] Mugwe J., Mugendi D., Mucheru-Muna M., Merckx R., Chianu J., Vanlauwe B. (2009). Determinants of the decision to adopt integrated soil fertility management practices by smallholder farmers in the central highlands of Kenya. Exp. Agric..

[16] Willy D.K., Holm-Müller K. (2013). Social influence and collective action effects on farm level soil conservation effort in rural Kenya. Ecol. Econ..

[17] De Souza Filho H.M., Young T., Burton M.P. (1999). Factors Influencing the Adoption of Sustainable Agricultural Technologies: Evidence from the State of Espıírito Santo, Brazil. Technol. Forecast. Soc. Chang..

[18] Mazvimavi K., Twomlow S. (2009). Socioeconomic and institutional factors influencing adoption of conservation farming by vulnerable households in Zimbabwe. Agric. Syst..

[19] Somda J., Nianogo A., Nassa S., Sanou S. (2002). Soil fertility management and socio-economic factors in crop-livestock systems in Burkina Faso: A case study of composting technology. Ecol. Econ..

[20] Costa N.B.d., Baldissera T.C., Pinto C.E., Garagorry F.C., de Moraes A., Carvalho P.C.d.F. (2019). Public policies for low carbon emission agriculture foster beef cattle production in southern Brazil. Land Use Policy.

[21] Paustian K. (2012). Agriculture, farmers and GHG mitigation: A new social network?. Carbon Manag..

[22] Sánchez B., Álvaro-Fuentes J., Cunningham R., Iglesias A. (2016). Towards mitigation of greenhouse gases by small changes in farming practices: Understanding local barriers in Spain. Mitig. Adapt. Strat. Glob. Chang..

[23] D’Emden F.H., Llewellyn R.S., Burton M.P. (2006). Adoption of conservation tillage in Australian cropping regions: An application of duration analysis. Technol. Forecast. Soc. Chang..

[24] Knowler D., Bradshaw B. (2007). Farmers’ adoption of conservation agriculture: A review and synthesis of recent research. Food Policy.

[25] Kragt M., Dumbrell N., Blackmore L. (2017). Motivations and barriers for Western Australian broad-acre farmers to adopt carbon farming. Environ. Sci. Policy.

[26] Wandel J., Smithers J. (2009). Factors affecting the adoption of conservation tillage on clay soils in southwestern Ontario, Canada. Am. J. Altern. Agric..

[27] Liu Y., Ruiz-Menjivar J., Zhang L., Zhang J., Swisher M.E. (2019). Technical training and rice farmers’ adoption of low-carbon management practices: The case of soil testing and formulated fertilization technologies in Hubei, China. J. Clean. Prod..

[28] Moges D.M., Taye A.A. (2017). Determinants of farmers’ perception to invest in soil and water conservation technologies in the North-Western Highlands of Ethiopia. Int. Soil Water Conserv. Res..

[29] Li C., Ting Z., Rasaily R.G. (2010). Farmer’s Adaptation to Climate Risk in the Context of China. Agric. Agric. Sci. Procedia.

[30] Field C., Barros V., Change I. (2014). Climate Change 2014: Impacts, Adaptation, and Vulnerability.

[31] Woods B.A., Nielsen H.Ø., Pedersen A.B., Kristofersson D. (2017). Farmers’ perceptions of climate change and their likely responses in Danish agriculture. Land Use Policy.

[32] Deressa T.T., Hassan R.M., Ringler C., Alemu T., Yesuf M. (2009). Determinants of farmers’ choice of adaptation methods to climate change in the Nile Basin of Ethiopia. Glob. Environ. Chang..

[33] Nhemachena C., Hassan R.M. (2007). Micro-level analysis of farmers’ adaptation to climate change in Southern Africa. IFPRI Discuss. Pap..

[34] Bechtoldt M.N., Götmann A., Moslener U., Pauw W.P. (2021). Addressing the climate change adaptation puzzle: A psychological science perspective. Clim. Policy.

[35] Prokopy L.S., Arbuckle J.G., Barnes A.P., Haden V.R., Hogan A., Niles M.T., Tyndall J. (2015). Farmers and Climate Change: A Cross-National Comparison of Beliefs and Risk Perceptions in High-Income Countries. Environ. Manag..

[36] Simon winter Sustainable Intensification. https://www.syngentafoundation.org/sustainable-intensification.

[37] Azadi H., Moghaddam S.M., Burkart S., Mahmoudi H., Van Passel S., Kurban A., Lopez-Carr D. (2021). Rethinking resilient agriculture: From Climate-Smart Agriculture to Vulnerable-Smart Agriculture. J. Clean. Prod..

[38] Atanu S., Love H.A., Schwart R. (1994). Adoption of Emerging Technologies under Output Uncertainty. Am. J. Agric. Econ..

[39] Ngigi M.W., Mueller U., Birner R. (2017). Gender Differences in Climate Change Adaptation Strategies and Participation in Group-based Approaches: An Intra-household Analysis from Rural Kenya. Ecol. Econ..

[40] Tang J., Folmer H., Xue J. (2016). Adoption of farm-based irrigation water-saving techniques in the Guanzhong Plain, China. Agric. Econ..

[41] Tizale C.Y. (2007). The Dynamics of Soil Degradation and Incentives for Optimal Management in the Central Highlands of Ethiopia. Ph.D. Thesis.

[42] Gbetibouo G.A., Hassan R.M., Ringler C. (2010). Modelling farmers’ adaptation strategies for climate change and variability: The case of the Limpopo Basin, South Africa. Agrekon.

[43] Lewis B.D., Pattinasarany D. (2009). Determining Citizen Satisfaction with Local Public Education in Indonesia: The Significance of Actual Service Quality and Governance Conditions. Growth Chang..

[44] Vecchio Y., Agnusdei G.P., Miglietta P.P., Capitanio F. (2020). Adoption of Precision Farming Tools: The Case of Italian Farmers. Int. J. Environ. Res. Public Health.

[45] Mussida C., Zanin L. (2020). Determinants of the Choice of Job Search Channels by the Unemployed Using a Multivariate Probit Model. Soc. Indic. Res..

[46] Wooldridge J.M. (2003). Introductory Econometrics: A Modern Approach.

[47] Siddhartha C., Edward G. (1998). Analysis of multivariate probit models. Biometrika.

[48] Mahmood N., Arshad M., Mehmood Y., Faisal Shahzad M., Kächele H. (2021). Farmers’ perceptions and role of institutional arrangements in climate change adaptation: Insights from rainfed Pakistan. Clim. Risk Manag..

[49] Duan C., Ma X. (2011). Analysis on the intergenerational difference of Migrant workers in China. Labour Econ. Rev..

[50] National Bureau of Statistics of China (2016). Plan for the third National Agricultural Census.

[51] Mahmood N., Arshad M., Kaechele H., Shahzad M., Ullah A., Mueller K. (2020). Fatalism, Climate Resiliency Training and Farmers’ Adaptation Responses: Implications for Sustainable Rainfed-Wheat Production in Pakistan. Sustainability.

